# Draft Genome Sequence of Colistin-Resistant *Acinetobacter baumannii* Strain VB22595 Isolated from a Central Line-Associated Bloodstream Infection

**DOI:** 10.1128/genomeA.00835-16

**Published:** 2016-08-11

**Authors:** Balaji Veeraraghavan, Shalini Anandan, Naveen Kumar Devanga Ragupathi, Saranya Vijayakumar, Dhiviya Prabaa Muthuirulandi Sethuvel, Indranil Biswas

**Affiliations:** a Department of Clinical Microbiology, Christian Medical College, Vellore, Tamil Nadu, India; b Department of Microbiology, Molecular Genetics and Immunology, University of Kansas Medical Center, Kansas City, Kansas, USA

## Abstract

*Acinetobacter baumannii* is an important emerging pathogen that causes health care-associated infections. In this study, we determined the genome of a multidrug-resistant clinical strain, VB22595, isolated from a hospital in Southern India. The draft genome indicates that strain VB22595 encodes a genome of ~3.92 Mb in size and does not contain plasmid derived MCR-1 for colistin resistance.

## GENOME ANNOUNCEMENT

*Acinetobacter baumannii*, an opportunistic pathogen, is responsible for causing a wide range of infections including bacteremia, meningitis, ventilator-associated pneumonia, skin and soft tissue infections, and urinary tract infections (1). *A. baumannii* is intrinsically resistant to many antibiotics and it has the ability to acquire resistance determinants from the environment (2). Carbapenems are one of the drugs chosen to treat infections caused by multidrug-resistant *A. baumannii*. However, resistance to these agents is being increasingly reported worldwide (3).

In this report, we present the draft genome sequence of *A. baumannii* strain VB22595, isolated from a central line-associated bloodstream infection in a 12-year old male patient from Christian Medical College, India. Strain VB22592 was resistant to cefepime, piperacillin-tazobactam, imipenem, meropenem, aztreonam, amikacin, netilmicin, tobramycin, and colistin. When strain VB22595 was subjected to broth microdilution to determine the MIC for colistin, it was found to be >256 µg/ml. Since strain VB22595 was resistant to colistin, we decided to determine its whole-genome sequence (WGS).

WGS was performed on the Ion Torrent PGM platform using 400-bp read chemistry. Sequencing was done following the protocol recommended by Life Technologies. Raw reads were assembled *de novo* using AssemblerSPAdes software v4.4.0.1 in Torrent suite server version 4.4.3. Sequencing generated 228,653,931 bp sequences with 72 contigs (≥500 bp) of 57× coverage. The genome was annotated using the RAST (http://rast.nmpdr.org/rast.cgi) (4–6) and PATRIC (https://www.patricbrc.org) databases (7). The annotation revealed 3,860 CDS with eight ARDB and 54 CARD antimicrobial resistance genes. In addition, four and seven virulence factors have been identified by the VFDB and Victors databases, respectively (https://www.patricbrc.org).

The sequence type (ST) was found to be ST1305 (http://pubmlst.org/abaumannii/) based on the analysis of seven housekeeping genes included in the pubMLST database (*cpn60*-2, *gdhB*-3, *gltA*-1, *gpi*-96, *gyrB*-12, *recA*-2, and *rpoD*-3). Further analysis of antimicrobial resistance genes with Res Finder found different classes of resistance genes (8). These include beta-lactams [*bla*_OXA-23_, *bla*_OXA-66_, and *bla*_ADC-25_]; aminoglycosides [*strB, strA* and *armA*], sulfonamide [*sul2*], tetracycline [*tet (B)*], and macrolide [*mphE* and *msrE*]. WGS indicates that VB22595 does not carry a plasmid-encoded MCR-1 gene (9). Further transcriptomics analysis is needed to understand the mechanism behind the antibiotic resistance.

### Accession number(s).

The WGS of *A. baumannii* VB22595 was submitted to NCBI under the accession no. LTAJ00000000. The version described in this manuscript is version LTAJ01000000.
